# Tumor Microenvironment CD14^+^ Cells Correlate with Poor Overall Survival in Patients with Early-Stage Lung Adenocarcinoma

**DOI:** 10.3390/cancers14184501

**Published:** 2022-09-16

**Authors:** Erin L. Schenk, Jennifer M. Boland, Sarah G. Withers, Peggy A. Bulur, Allan B. Dietz

**Affiliations:** 1 Department of Medicine, Division of Medical Oncology, Univeristy of Colorado, Aurora, CO 80045, USA; 2 Department of Laboratory Medicine and Pathology, Mayo Clinic, Rochester, MN 55905, USA; 3 Human Cell Therapy Laboratory, Divisions of Transfusion Medicine and Experimental Pathology, Mayo Clinic, Rochester, MN 55905, USA

**Keywords:** lung cancer, tumor microenvironment, CD1

## Abstract

**Simple Summary:**

Lung cancer is the leading cause of cancer-related deaths worldwide in part due to high rates of recurrence even with early-stage disease. This suggests that new biomarkers are needed to improve predictions for patient outcomes and better understand the underlying biology that drives poor outcomes. We believe the immune cells within the tumor microenvironment (TME) are a key differentiator between patients who are cured and patients who experience lung cancer relapse. In this study, we investigated the presence of CD14^+^ cells within the lung adenocarcinoma TME through immunohistochemistry and found that higher levels of CD14^+^ cells were correlated with shorter patient survival. In vitro studies revealed a bi-directional cross-talk between CD14^+^ cells and lung cancer that improved lung cancer cell recovery after chemotherapy. Our observations suggest that TME CD14^+^ infiltration is a prognostic marker in lung cancer and that further studies are needed to understand CD14^+^-cell-mediated mechanisms that influence patient outcomes.

**Abstract:**

Patients with early-stage lung adenocarcinoma have a high risk of recurrent or metastatic disease despite undergoing curative intent therapy. We hypothesized that increased CD14^+^ cells within the tumor microenvironment (TME) could stratify patient outcomes. Immunohistochemistry for CD14 was performed on 189 specimens from patients with lung adenocarcinoma who underwent curative intent surgery. Outcomes and associations with clinical and pathologic variables were determined. In vitro studies utilized a coculture system to model the lung cancer TME containing CD14^+^ cells. Patients with high levels of TME CD14^+^ cells experienced a median overall survival of 5.5 years compared with 8.3 and 10.7 years for those with moderate or low CD14 levels, respectively (*p* < 0.001). Increased CD14^+^ cell tumor infiltration was associated with a higher stage at diagnosis and more positive lymph nodes at the time of surgery. This prognostic capacity remained even for patients with early-stage disease. Using an in vitro model system, we found that CD14^+^ cells reduced chemotherapy-induced cancer cell death. These data suggest that CD14^+^ cells are a biomarker for poor prognosis in early-stage lung adenocarcinoma and may promote tumor survival. CD14^+^ cell integration into the lung cancer TME can occur early in the disease and may be a promising new therapeutic avenue.

## 1. Introduction

Lung cancer is the leading cause of cancer-related mortality in the United States, accounting for over 150,000 deaths each year [1]. Unfortunately, even patients with early-stage non-small cell lung cancer (NSCLC) who undergo curative-intent therapy have a significant risk of recurrence, ranging from 5% to 19% for stage I disease up to 40% in patients with stage III disease [2]. This underscores the acute need to identify new biomarkers not only for improved prognostication but also to better understand the underlying biology that drives poor outcomes.

Within the tumor microenvironment (TME), the immune system has emerged as a therapeutic and prognostic tool in cancer [3]. In NSCLC, multiple studies have demonstrated a relationship between TME immune markers and patient outcomes [4,5]. The impact of TME lymphocyte populations is consistent with the broader role of each cell subset [4,5]. While tumor recurrence can be prevented by the adaptive arm of the immune system, innate immune cells, such as myeloid cells, can adopt a phenotype that supports tumor survival. In animal models of lung cancer, myeloid cell accumulation within the TME promotes angiogenesis, bolsters an immunosuppressive environment, and ultimately drives tumor progression [6,7,8,9]. In patients with NSCLC, the impact of TME myeloid cells is incompletely understood, as studies with large cohorts using tumor-associated macrophage markers CD68 or CD163 or the neutrophil marker CD66 report mixed results [4,5]. Consequently, clearly defining the impact of TME myeloid cells on NSCLC patient outcomes has been challenging.

One myeloid marker that has not been well-characterized is CD14, which classically identifies a heterogeneous population of mononuclear phagocytes with significant functional plasticity. We and others have reported the tumor-driven phenotypic and functional changes in CD14^+^ cells after coculture [10,11,12,13]. Even with those CD14^+^ cells isolated from healthy volunteers, tumors robustly promote a CD14^+^HLA-DR^lo/neg^ phenotype, which is broadly defined as a monocytic myeloid-derived suppressor cell. This in vitro model of the cancer TME generates CD14^+^HLA-DR^lo/neg^ cells with decreased capacity to mature into dendritic cells, elaborate proangiogenic factors, and potently inhibit T-cell proliferation, similar to what is observed with cancer-patient-derived CD14^+^HLA-DR^lo/neg^ cells [11,12,13]. Clinically, an increasing burden of peripheral-blood-circulating CD14^+^HLA-DR^lo/neg^ cells correlates with poor patient outcomes across multiple tumor types, including NSCLC [10,11,14,15,16,17,18,19]. Recently, the higher levels of TME CD14^+^ cells were found to be associated with reduced survival in early-stage squamous NSCLC [20]. While collectively, these studies suggest that CD14^+^ cells within the TME of patients with lung adenocarcinoma may contribute to poor outcomes, little is known about their prognostic impact. A better understanding of how TME CD14^+^ cells affect patient survival could improve risk stratification and support the development of new therapeutic targets. Here, through immunohistochemistry (IHC), we established that CD14^+^ cells are present within the adenocarcinoma NSCLC TME and are prognostic for patient outcomes. Additionally, in vitro, we approximated the lung cancer TME containing CD14^+^ cells and demonstrated that CD14^+^ cells reduce chemotherapy-induced cancer cell death.

## 2. Materials and Methods

### 2.1. Immunohistochemistry

Formalin-fixed paraffin-embedded tissue blocks were sectioned into 5 µm slides and stained by the Pathology Research Core (Mayo Clinic, Rochester, MN, USA). IHC was performed using anti-CD14 (Ventana, clone EPR3653), and semi-quantitative scoring was performed by J.M.B, as previously described [12]. The number of CD14^+^ cells was scored as low (only rare CD14^+^ cells observed, occupying <5% of the tumor surface area), moderate (CD14^+^ cells occupying approximately 5–20% of the tumor surface area), or high (CD14^+^ cells occupying >20% of the tumor surface area).

### 2.2. Clinical Data

This study was approved by the Mayo Clinic Institutional Review Board. Written informed consent was obtained from all patients in accordance with the Declaration of Helsinki. The patients were identified from the Mayo Clinic Lung Cancer Repository who underwent curative surgical resection of lung adenocarcinoma between 2004 and 2007 and had available residual tumor specimens. Their electronic medical record was reviewed, and the pertinent clinical data including age at the time of surgery, gender, smoking status, pack years, and the last follow-up date or date of death was extracted. The patients were staged based on surgical findings using the 8th edition of the AJCC TNM system for NSCLC [21]. For overall survival, the patients were censored at the date of their last follow-up. For cancer-specific survival, the patients were censored at the date of their last follow-up or death from causes other than lung cancer.

### 2.3. Lung Cancer Cell Lines

Lung cancer cell lines were purchased from ATCC and maintained in recommended growth media RPMI (Corning, Corning, NY, USA) + 10% fetal bovine serum (Corning). The purchased cell lines were stably transfected with nuclear restricted GFP with CellPlayer NucLight Green (Essen Bioscience, Ann Arbor, MI, USA) and selected as per the manufacturer’s protocol. GFP expression was validated via flow cytometry and reselected if GFP expression was <95%. The cell lines were tested every 3 months for mycoplasma.

### 2.4. CD14^+^ Cell Isolation

CD14^+^ cells were purified from the peripheral blood of healthy volunteers as previously described [12]. Briefly, after the isolation of the peripheral blood mononuclear cells, CD14^+^ cells were positively selected with CD14 MicroBeads (Miltenyi Biotec, North Rhine-Westphalia, Germany) and isolated via an AutoMacs Cell Separator (Miltenyi Biotech). Freshly isolated CD14^+^ cells were used for each experiment.

### 2.5. CD14^+^ Cell Migration

Lung cancer cell lines were plated at a density of 10^6^ cells/mL, and supernatants were collected after 3 days. The supernatants were filtered with 0.2 µm membrane and stored at −20 °C. The isolated CD14^+^ cells were fluorescently labeled with CellTracker Green Dye (Life Technologies, Carlsbad, CA, USA) and 10^5^ labeled cells were placed in the upper chamber of 5 µm pore transwell inserts (Corning). Warmed supernatants from lung cancer cell lines were placed in the bottom chamber. Real-time phase and fluorescence live cell imaging was used to follow CD14^+^ cell migration, and object counts were obtained every 2 h (Essen Bioscience, IncuCyte FLR).

### 2.6. HLA-DR Expression

CD14^+^ cell HLA-DR expression was evaluated via flow cytometry on the day of isolation and 48 h after coculture. Briefly, cells were washed and stained with anti-CD14 (eBioscience, Clone 61D3, San Diego, CA, USA); then, a LIVE/DEAD™ Fixable Violet viability dye was added (ThermoFisher, Waltham, MA, USA), as per the manufacturer’s recommendations. The cells were fixed with a 4% paraformaldehyde solution and run on a Beckman Coulter Gallios flow cytometer.

### 2.7. Coculture Experiments

Lung cancer cell lines and isolated CD14^+^ cells were cocultured in a 1:3 ratio in RPMI and 1% FBS in 24-well plates. All coculture conditions were plated in triplicate. For experiments with chemotherapy, after 48 h of culture with or without CD14^+^ cells, 1 µm cisplatin was added to each well. Twenty-four hours later, the wells were washed twice with PBS, and fresh media were replaced. Flow cytometry for viable tumor cells was performed using anti-CD14 and viability dye as mentioned above starting the day after cisplatin exposure and on subsequent days as described. Each individual sample was run for 2 min on the flow cytometer. The viable tumor cells were quantified based on viable GFP^+^ cells. Data analysis was performed with Kaluza Software v2.1 (Beckman Coulter, Brea, CA, USA). Data represent the average and standard deviation of 3 experimental replicates.

### 2.8. Data Analysis

Kaplan–Meier curves were generated with Prismv9.40 (GraphPad, San Diego, CA, USA) and analyzed with a log-rank test. Unpaired *t*-tests and ANOVA analyses were performed with Prismv9.40 (GraphPad). Cox proportional hazards models were calculated with JMPv14.0.0 (SAS, Stockholm, Sweden).

## 3. Results

### 3.1. CD14^+^ Cell Tumor Infiltration Associates with Shorter Overall Survival in Patients with Early-Stage Lung Adenocarcinoma

We identified 189 patients with lung adenocarcinoma and available tissue from the Mayo Clinic Lung Cancer Repository who underwent curative-intent resection between 2004 and 2007 (Table 1).

**Figure 1:**
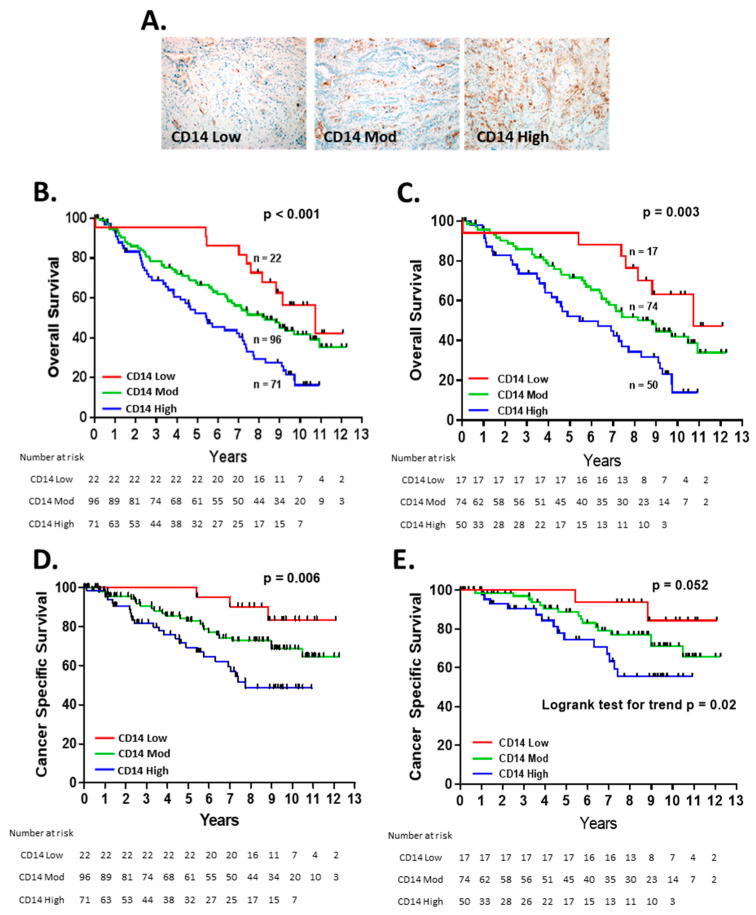
CD14^+^ cell infiltration in the NSCLC TME predicts overall survival: (**A**) representative images of CD14 low, moderate, and high. Kaplan–Meier curves of overall survival in all patients (n = 189) (**B**) and stage I and IIA patients (n = 141) (**C**) and cancer-specific survival for all patients (**D**) stage I and IIA patients (**E**). Patients were censored at date of last follow-up or non-lung-cancer-related death.

The IHC for CD14 expression demonstrated that all the tumor specimens contained CD14^+^ cells. The tumors were classified into three groups based on a low, moderate, or high frequency of CD14^+^ cells (Figure 1A). The level of CD14^+^ cell TME infiltration was strongly associated with the overall survival (OS) (*p* < 0.001) (Figure 1B). Patients with a high level of TME CD14^+^ cells experienced a median OS of 5.5 years, compared with 8.3 years and 10.7 years for those with moderate or low CD14^+^ levels, respectively.

For patients with early-stage (stage I and IIA) NSCLC, outcomes can vary significantly, from cure to disease relapse and death, and, in our cohort, the patients with the early-stage disease were present in all three of the CD14 TME groups [21]. Within the subgroup of patients with early-stage disease, TME CD14 levels were significantly associated with OS (*p* = 0.003) (Figure 1C), suggesting that this marker can provide prognostic information at an early stage. CD14 status was associated with cancer-specific survival for the entire cohort (Figure 1D) and trended towards significance for early-stage patients (Figure 1E). In multivariate analysis, TME CD14^+^ cell infiltration was an independent risk factor for OS in the entire patient cohort and patients with early-stage disease (Table 2).

**Figure 2:**
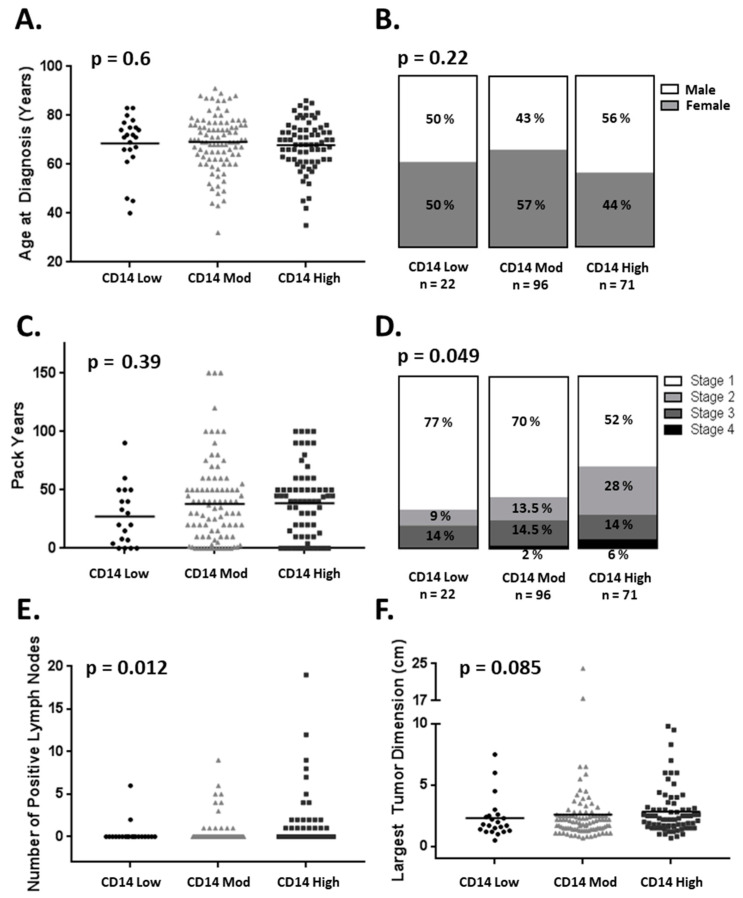
CD14^+^ cell infiltration association with clinical and pathologic variables. CD14^+^ cell infiltration is not associated with (**A**) age at diagnosis (**B**) gender or (**C**) pack years. CD14^+^ cell infiltration is associated with (**D**) advanced stage at diagnosis and (**E**) positive lymph nodes found at time of surgery but not (**F**) largest dimension of the primary tumor (one-way ANOVA).

For all the patients, the level of CD14^+^ TME cells identified patient cohorts independently of the known prognostic factors of age, gender, or smoking pack years (Figure 2A–C) [22,23]. The increasing levels of TME CD14^+^ cells were associated with a higher stage at diagnosis and the number of positive lymph nodes on pathologic evaluation (Figure 2D,E) but not the largest tumor dimension (Figure 2F).

### 3.2. CD14^+^ Cells Are Recruited and Altered by Lung Cancer Cell Lines

**Figure 3:**
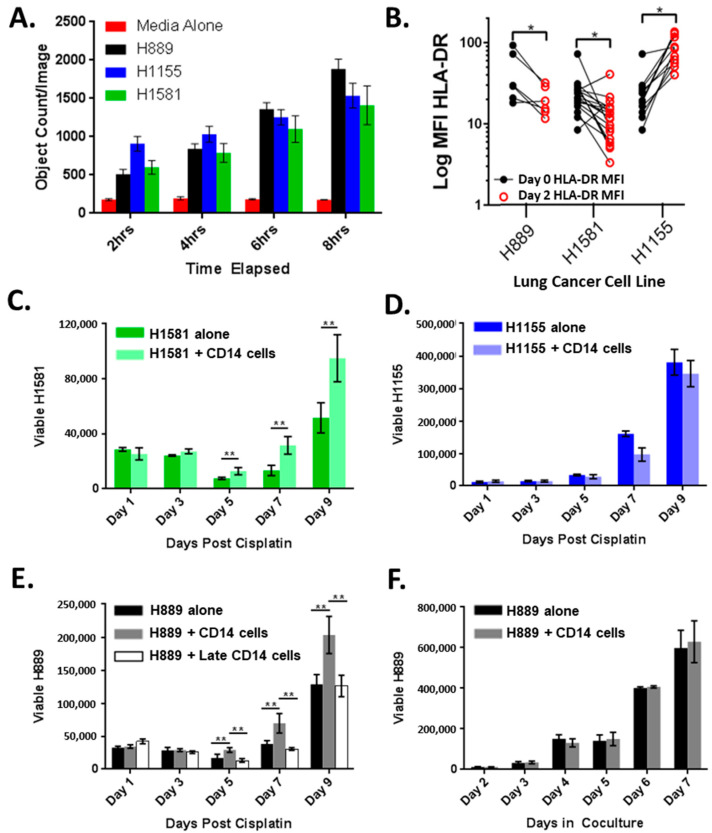
Cross-talk between lung cancer cells and CD14^+^ cells: (**A**) CD14^+^ cell migration in response to cell-free tumor supernatants quantified by fluorescent live cell imaging; (**B**) CD14^+^ cell HLA-DR expression before and after 48 h coculture with lung cancer cell lines. Coculture with CD14^+^ cells results in improved recovery of (**C**) H1581 but not (**D**) H1155; (**E**) H889 cocultured with CD14^+^ cells for 48 h results in improved tumor recovery but not if CD14^+^ cells were added just before chemotherapy (H889 + Late CD14 cells); (**F**) growth of H889 alone or in coculture with CD14^+^ cells. * *p* < 0.05, ** *p* < 0.01 by 2 sided *t*-test.

Since increasing CD14^+^ cells in the lung adenocarcinoma TME was associated with shortened OS and more positive lymph nodes at the time of surgical resection, we developed an in vitro coculture model system to study the interactions between lung cancer cell lines and CD14^+^ cells. The cell line supernatants stimulated a robust CD14^+^ cell migration across the transwell membranes compared with culture media alone (Figure 3A). At the baseline, the lung cancer cell lines were releasing detectable levels of CCL2, a potent chemokine for CD14^+^ cells (Supplemental Figure S1). CD14^+^ cell coculture with lung cancer cell lines H889 and H1581 resulted in CD14^+^ cell HLA-DR downregulation, a phenotype observed in other models, while the coculture with H1155 did not (Figure 3B).

### 3.3. CD14^+^ Cells Reduce Chemotherapy-Induced Tumor Cell Death

Based on these data, we hypothesized that lung cancer cell lines retain the ability to recruit CD14^+^ cells for a survival advantage. Chemotherapy resistance is a major clinical challenge, and we tested if CD14^+^ cells reduced tumor sensitivity to cisplatin (Figure 3C–E). H1581 and H889 (cisplatin IC50 of 3 and 2.4 μM, respectively) cocultured with CD14^+^ cells for 48 h prior to cisplatin exposure demonstrated an accelerated recovery after a cisplatin challenge (Figure 3C,E), though this was not observed in H1155 (cisplatin IC50 1 μM) (Figure 3D) [24,25]. Promoting tumor recovery appears to be a shared trait of CD14^+^ cells, as multiple unique healthy donor CD14^+^ cells were able to improve lung cancer recovery (Figure 3C,E). Importantly, CD14^+^ cells added to coculture shortly before cisplatin exposure did not result in improved tumor recovery in H889 (Figure 3E) or in H1581 (Supplemental Figure S2). In addition, under normal culture conditions, the presence of CD14^+^ cells did not alter tumor growth kinetics (Figure 3F). These data suggest that improved tumor recovery after chemotherapy exposure requires a period of cross-talk between CD14^+^ cells and tumor cells and is not due to a reduction in chemotherapy exposure or increased cancer cell proliferation over the baseline.

## 4. Discussion

Molecular and immune biomarkers have improved the treatment landscape for NSCLC by pairing patients with the therapies most likely to result in tumor response. For those patients with early-stage NSCLC, these biomarkers have started to shape clinical decision making [26]. However, despite the application of these biomarkers, significant variability exists in the degree of benefit in patients’ experience, suggesting that further refinement is needed. In this study, we found that CD14^+^ cell TME infiltration through IHC identified the cohorts of patients with distinct outcomes after surgical resection, a mainstay of most curative intent therapy for NSCLC. The prognostic value of CD14^+^ TME status was underscored by the median survival of 5.5 years for early-stage patients with high levels of infiltration versus 10.7 years for low levels of CD14^+^ cell infiltration in our cohort. This observation is consistent with the outcomes for patients with squamous cell lung cancer, which follow a similar pattern [20]. In 380 patients with predominantly early-stage squamous lung cancer, a higher TME expression of CD14 detected through RNA-seq and IHC was associated with shorter overall survival [20]. This association with survival is likely, in part, related to the observation that TME myeloid cells promote tumor spread. In the murine models of NSCLC, the monocytes within the squamous cell lung cancer TME directed the remodeling of the extracellular matrix that was necessary for tumor metastases [20]. Consistent with this, in the patient cohort with squamous cell lung cancer, the higher levels of CD14 TME expression were correlated with post-operative disease recurrence [20]. Similarly, in our patient cohort with lung adenocarcinoma, the higher levels of CD14^+^ TME infiltration were associated with a higher number of tumor-bearing lymph nodes at the time of surgery and shorter lung cancer-specific survival. Taken together, our data and those of others suggest that the presence of TME CD14^+^ cells may identify those patients more at risk for aggressive disease.

Classically, CD14^+^ cells within the TME are believed to impact patient outcomes by inhibiting T-cell responses [27]. Within the lung cancer TME, CD14^+^ cells may also influence T-cell responses by expressing immunosuppressive T-cell checkpoints, as was noted in a subset of 61 patients with early and more advanced stage resected NSCLC of either histology. In those patients, the higher levels of CD14^+^HLA-DR^lo/neg^B7-H3^+^ TME cells correlated with a shorter relapse-free survival rate [28]. We observed HLA-DR downregulation on CD14^+^ cells after coculture with lung cancer cell lines, suggesting an immunosuppressive phenotype, as observed in patients with advanced lung cancer and other tumor types [11,18,29,30]. However, in our model system, cancer cell outgrowth was related to the cross-talk with CD14^+^ cells. Recently, data have emerged indicating that generating local immunosuppression may not be the initial event connecting CD14^+^ TME cells with poor patient outcomes in early-stage lung cancer [31]. The CD14^+^ cells freshly isolated from most lung tumor resections did not inhibit autologous T-cell proliferation or INFγ production and only a minority of the patients’ specimens contained the increased levels of CD14^+^ cells that blocked a T-cell response [31]. These data suggest that the relationship between lung cancer progression and the TME is complex, and non-immunosuppressive mechanisms may predominate the tumor-promoting role of CD14^+^ cells in the early lung cancer TME.

One emerging mechanism of CD14^+^-cell-mediated tumor survival, not related to immunosuppression, is improved tumor resistance to local environmental stresses, such as chemotherapy or other systemic therapy. Previously, CD14^+^HLA-DR^lo/neg^ cells isolated from lung cancer patients cocultured with a lung cancer cell line resulted in an increased number of viable tumor cells 4 days after a chemotherapy challenge [32]. These coculture systems provide an opportunity to directly assess the CD14^+^-cell-mediated, transcriptional changes acquired by the cancer cell and is a model for exploring the upregulated pathways that potentially impart chemotherapy resistance [33]. While chemotherapy has been a mainstay of curative intent therapy for resectable lung cancer, recent advances now include targeted therapy or immunotherapy for select groups of patients [26]. Other groups have identified that CD14^+^ cells imparted targeted therapy resistance to lung cancer cell lines and that an increasing CD14^+^ cell presence in the TME is associated with a shorter duration of patient benefit with targeted therapy [34]. In a murine model of lung cancer, the inhibition of the gamma isoform of phosphoinositide 3-kinase in myeloid cells improved tumor sensitivity to immunotherapy [35]. In patients with metastatic NSCLC, the epigenetic analysis identified a signature that correlated with progression-free survival on anti-PD-1 therapy [36]. Notably, the tumors with a negative epigenetic signature were enriched with myeloid cells [36]. Collectively, these data suggest that TME CD14^+^ cell infiltration could inform outcome expectations for patients with lung cancer across treatment paradigms.

This study has some limitations and has raised a number of questions still to be answered. The functional diversity of the myeloid cells within the lung cancer TME is not well-characterized by a single IHC marker. A more in-depth exploration of cell surface markers and transcription factors could better delineate which CD14^+^ myeloid cell subset is prognostic for patient outcomes. Further work developing CD14 as a prognostic biomarker in early-stage lung cancer will be strengthened by using an external validation cohort, but the similarities between our data and those of the previously published squamous cohort are encouraging [20]. Based on the data reported by our group and others, the presence of CD14^+^ cells within the TME may represent a biomarker for patients with lung cancer across therapeutic approaches and could represent a shared avenue for future therapeutic investigation. However, one limitation of our data is that the majority of the patients underwent surgical resection with no other systemic therapy. While this remains the current standard of care for patients with stage I disease, a more modern cohort reflecting current practice patterns would better evaluate CD14^+^ cells as a biomarker in the context of neoadjuvant and adjuvant chemoimmunotherapy approaches [24]. Furthermore, patient outcomes in our cohort could be influenced by the presence of driver oncogenes but these data were not available for this cohort. While CD14^+^ cells within the TME may be a common target across patients and treatments, the full breadth of mechanisms that drive tumor survival through cross-talk with CD14^+^ cells remains an active area of investigation. Additionally, within the TME, stromal cells may also benefit from cross-talk with CD14^+^ cells, and further work is necessary to define this interaction. Overall, our data support the hypothesis that the increasing levels of CD14^+^ cell infiltration in early-stage lung adenocarcinoma result in a tumor survival benefit that associates with poor patient outcomes. Gaining a better understanding of how and which phenotype of CD14^+^ cells influence tumor survival may help personalize the currently available lung cancer therapy and drive new therapeutic strategies.

## 5. Conclusions

In conclusion, we demonstrated that the degree of CD14^+^ cell TME infiltration was associated with survival in 189 patients with resected lung adenocarcinoma. Notably, this association remained significant in patients with early-stage disease, suggesting that even in early stages, the recruitment of CD14^+^ cells into the TME is a significant event that can alter the expectations for patient outcomes. Furthermore, we demonstrated that the cross-talk between CD14^+^ cells and lung cancer cell lines resulted in CD14^+^ cell recruitment, the downregulation of CD14^+^ cell HLA-DR, and improved cancer cell recovery after chemotherapy exposure. Together, these data highlight the variety of the potential avenues through which CD14^+^ cells within the TME may promote tumor cell survival. In conjunction with the previously published data, we believe the presence of CD14^+^ cells within the lung cancer TME may represent a prognostic marker that could inform treatment expectations across histologies and treatment approaches. This shared observation underscores the need to validate TME CD14^+^ infiltration as a prognostic marker in lung cancer, and for further studies to identify the immune and non-immune mechanisms by which CD14^+^ cells influence patient outcomes.

## Figures and Tables

**Table 1:** Patient characteristics.

**Age (years)**	
Median	70
Range	32–91
**Gender**	
F	97
**Smoking Status**	
Never	32
Current/former	157
Median pack/years	35
Range	1–150
**Surgical Procedure**	
Lobectomy	122
Lobectomy + wedge	12
Segmentectomy	6
Segmentectomy + wedge	1
Wedge alone	42
Pneumonectomy	5
Biopsy	1
**Lymph Nodes Resected**	
Median	19
Range	0–52
**Pathological Stage**	
IA	81
IB	40
IIA	20
IIB	16
IIIA	24
IIIB	2
IV	6
**Systemic Therapy**	
Chemotherapy	28
Neoadjuvant chemotherapy	3

**Table 2:** Univariate and Multivariate Analysis for Patient Overall Survival.

	Univariate Analysis All Patients		Multivariate Analysis All Patients	
	Hazard Ratio (95% CI)	*p* Value	Hazard Ratio (95% CI)	*p* Value
**Gender**				
F	1		1	
M	1.67 (1.15–2.44)	0.007	1.89 (1.19–2.78)	0.006
**Age at Diagnosis**				
<70 years	1		1	
≥70 years	1.70 (1.23–2.38)	0.0011	1.98 (1.31–3.00)	0.001
**CD14 IHC Score**				
1	1		1	
2	1.89 (1.01–4.01)	0.045	2.74 (1.21–6.23)	0.016
3	3.07 (1.66–6.5)	<0.0001	3.71 (1.59–8.68)	<0.003
**Stage at Diagnosis**				
1	1		1	
2	1.15 (0.78–1.68)	0.46	0.80 (0.40–1.59)	0.52
3	1.65 (1.16–2.36)	0.006	0.63 (0.24–1.66)	0.35
4	2.15 (1.2–3.62)	0.012	3.31 (0.90–12.14)	0.071
**Surgical Procedure**				
Lobectomy	1		1	
Wedge	1.02 (0.65–1.61)	0.92	1.84 (1.06–3.19)	0.029
Segmentectomy	1.37 (0.56–3.39)	0.49	3.11 (1.17–8.21)	0.023
Pneumonectomy	4.23 (1.30–13.72)	0.016	4.20 (1.12–15.75)	0.036
**Largest T Dimension**				
≤3 cm	1		1	
>3cm–≤7 cm	1.43 (0.97–2.07)	0.067	1.6 (0.92–2.79)	0.1
>7 cm	5.47 (3.13–9.07)	<0.0001	11.39 (3.68–35.25)	<0.001
**Number Positive Lymph Nodes**				
0	1		1	
≥1	1.79 (1.26–2.53)	0.001	1.99 (0.91–4.40)	0.09
**Smoking Status**				
<35 pack years	1		1	
≥35 pack years	1.47 (1.15–1.91)	0.002	1.41 (0.91–2.19)	0.12
	**Univariate Analysis Early-Stage Patients**		**Multivariate Analysis Early-Stage Patients**	
	**Hazard Ratio (95% CI)**	***p* Value**	**Hazard Ratio (95% CI)**	***p* Value**
**Gender**				
F	1		1	
M	1.65 (1.06–2.56)	0.027	1.73 (1.07–2.80)	0.024
**Age at Diagnosis**				
<70 years	1		1	
≥70 years	2.05 (1.31–3.20)	0.002	2.38 (1.47–3.86)	<0.001
**CD14 IHC Score**				
1	1		1	
2	1.80 (0.81–4.02)	0.15	3.04 (1.16–7.94)	0.023
3	3.27 (1.44–7.41)	0.005	5.08 (1.86–13.91)	0.002
**Surgical Procedure**				
Lobectomy	1		1	
Wedge	1.13 (0.67–1.90)	0.65	1.45 (0.81–2.89)	0.21
Segmentectomy	0.91 (0.29–2.92)	0.88	2.23 (0.66–7.50)	0.19
Pneumonectomy	13.28 (1.69–104.28)	0.014	23.44 (2.78–197.88)	0.004
**Largest T Dimension**				
≤3 cm	1		1	
>3 cm–≤4 cm	1.46 (0.70–3.03)	0.32	1.27 (0.58–2.76)	0.55
>5 cm	1.19 (0.48–2.96)	0.70	0.70 (0.21–2.36)	0.56
**Stage at Diagnosis**				
1	1		1	
2	1.50 (0.84–2.66)	0.17	1.53 (0.71–3.29)	0.42
**Smoking Status**				
<35 pack years	1		1	
≥35 pack years	1.27 (0.80–2.0)	0.30	1.22 (0.75–1.98)	0.42

## Data Availability

The data that support the findings of this study are available from the corresponding author, E.L.S., upon reasonable request.

## Supplementary Materials

The following supporting information can be downloaded at: https://www.mdpi.com/article/10.3390/cancers14184501/s1, Figure S1: Lung cancer cell lines secrete CCL2; Figure S2: H1581 cocultured with CD14^+^ cells for 48 h results in improved tumor recovery but not if CD14^+^ cells were added just before chemotherapy (HH1581 + Late CD14^+^ cells).
