# Supplementary material for: Tumor Microenvironment CD14+ Cells Correlate with Poor Overall Survival in Patients with Early-Stage Lung Adenocarcinoma
Source: Cancers (Basel). 2022 Sep 16;14(18):4501. doi: 10.3390/cancers14184501 (PMC9496975; doi:10.3390/cancers14184501)
Supplement: Supplementary file 1 [file cancers-14-04501-s001.zip › cancers-1862370-supplementary.pdf]

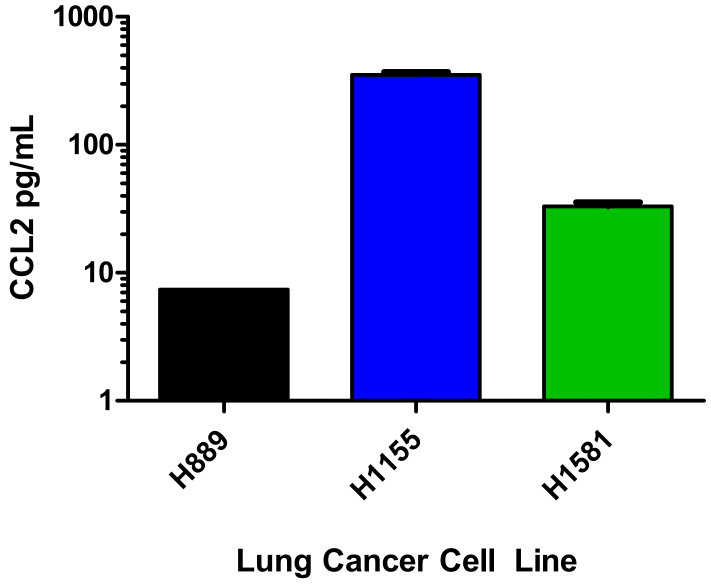

Supplementary Figure S1. Lung cancer cell lines secrete CCL2. For each cell line, 10^6 cells were cultured under normal culture conditions for 72 hours. Cell free supernatants were harvested and analyzed for CCL2 by ELISA (R&D Systems). Bars represent average of 2 readings ± SD.

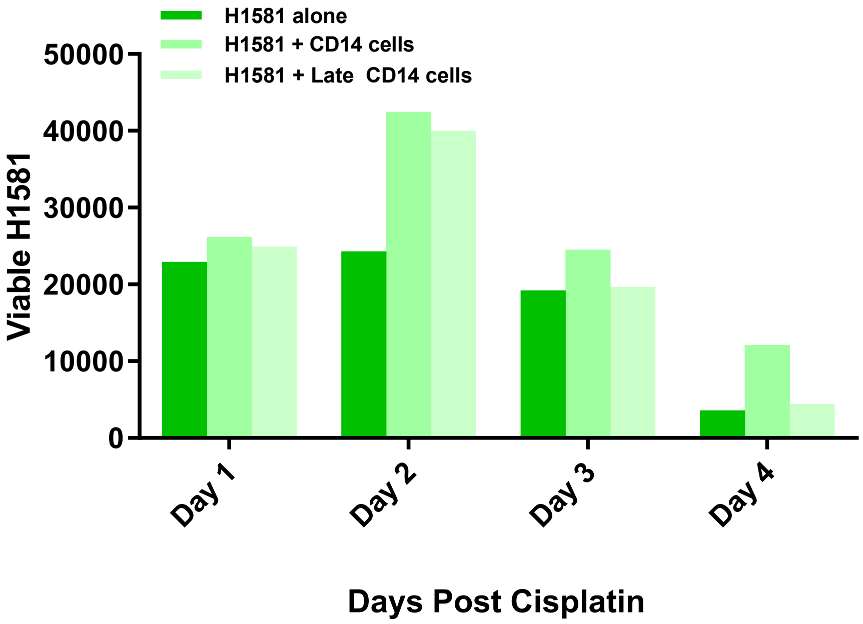

Supplementary Figure S2. H1581 cocultured with $CD14^{+}$ cells for 48 hours results in improved tumor recovery but not if $CD14^{+}$ cells were added just before chemotherapy (HH1581 + Late $CD14^{+}$ cells).
